# The 6-minute walk test predicts mortality in a pulmonary nontuberculous mycobacteria-predominant bronchiectasis cohort

**DOI:** 10.1186/s12879-022-07054-6

**Published:** 2022-01-21

**Authors:** Rebekah A. Blakney, Emily E. Ricotta, Dean Follmann, Jessica Drew, Kelly A. Carey, Lisa N. Glass, Chevalia Robinson, Sandra MacDonald, Pamela J. McShane, Kenneth N. Olivier, Kevin Fennelly, D. Rebecca Prevots

**Affiliations:** 1 Epidemiology and Population Studies Unit, Laboratory of Clinical Immunology and Microbiology, Division of Intramural Research, National Institute of Allergy and Infectious Diseases (NIAID), National Institutes of Health, Bethesda, MD USA; 2 Biostatistics Research Branch, Division of Clinical Research, NIAID, Bethesda, USA; 3 Pulmonary, Critical Care, and Sleep Disorders Division, George Washington University School of Medicine and Health Sciences, Washington, DC USA; 4 Laboratory of Chronic Airway Infection, Pulmonary Branch, Division of Intramural Research, National Heart, Lung, and Blood Institute, National Institutes of Health, MD Bethesda, USA; 5 University of Texas Health Science Center at Tyler, Tyler, TX USA; 6 5601 Fishers LN, RM 7D10, Rockville, MD 20852 USA

**Keywords:** Bronchiectasis, Nontuberculous mycobacteria, Mortality, Patient reported outcomes, Health-related quality of life, 6-Minute walk test

## Abstract

**Background:**

Bronchiectasis is a chronic lung condition frequently associated with nontuberculous mycobacteria pulmonary (NTM) disease. Persons with these conditions are at increased risk of mortality. Patient reported outcome (PRO) instruments and the 6-minute walk test (6MWT) have been shown to predict mortality for several lung conditions, but these measures have not been fully evaluated for bronchiectasis and NTM.

**Methods:**

We conducted a retrospective cohort study among adult patients enrolled in a natural history study of bronchiectasis at the National Heart, Lung, and Blood Institute. Electronic medical records were queried for demographic, clinical, microbiologic, radiographic, and PRO instrument data: St. George’s Respiratory Questionnaire (SGRQ), Medical Research Council Dyspnea Scale, and the Pulmonary Symptom Severity Score (PSSS). The study baseline date was defined as the patient’s first visit after January 1st, 2015 with a SGRQ or 6MWT completed. Follow-up was defined as the interval between the study baseline visit and date of death or December 31st, 2019. Sex-stratified Cox proportional-hazards regression was conducted to identify predictors of mortality. Separate models were run for each PRO and 6MWT measure, controlling for age, body mass index (BMI), fibrocavitary disease status, and *M. abscessus* infection.

**Results:**

In multivariable Cox proportional-hazards regression models, the PSSS-severity (aHR 1.29, 95% CI 1.04–1.59), the 6MWT total distance walked (aHR 0.938, 95% CI 0.896–0.981) and distance saturation product (aHR 0.930, 95% CI 0.887–0.974) independently predicted mortality. In addition, BMI was significantly predictive of mortality in all models.

**Conclusions:**

The 6MWT and a PRO instrument capturing symptom severity are independently predictive of mortality in our cohort of bronchiectasis patients.

**Supplementary Information:**

The online version contains supplementary material available at 10.1186/s12879-022-07054-6.

## Background

Bronchiectasis is a chronic pulmonary condition characterized by chronic inflammation and dilation of the bronchi, which results in significant morbidity due to recurrent respiratory infections and impaired lung function. Bronchiectasis is associated with an increased risk of mortality compared to healthy controls [1]. Persons with bronchiectasis are at very high risk of chronic infection with nontuberculous mycobacteria (NTM) [2], which is also associated with an increased risk of mortality even after controlling for comorbidities [3–5]. A recent meta-analysis of five-year mortality among persons with *Mycobacterium avium* complex (MAC) lung disease identified several consistent predictors of mortality including male sex, fibrocavitary disease, and the presence of comorbidities. Several studies also found low body mass index (BMI) to be a risk factor for increased mortality [6]. Functional measures such as the 6-Minute Walk Test (6MWT) have been less frequently measured systematically for studies of NTM and bronchiectasis disease progression. However, these measures have been identified as important mortality predictors for chronic obstructive pulmonary disease (COPD) [7] as well as for idiopathic pulmonary fibrosis [8]. Furthermore, the 6MWT correlates with quality-of-life measurements in patients with bronchiectasis and MAC lung disease [9, 10].

The Food and Drug Administration has urged the incorporation of patient reported outcome (PRO) instruments into clinical trials and a public meeting with representation of pulmonary NTM patients, most of whom had underlying bronchiectasis, highlighted the importance of quality of life and symptom measures [11, 12]. PRO instruments measuring health-related quality of life (HRQL) include the St. George’s Respiratory Questionnaire (SGRQ), which is widely used in COPD, and has been associated with mortality in COPD patients [13]. The SGRQ has also been validated for bronchiectasis [14]. PRO instruments capturing symptoms and symptom burden include the Medical Research Council (MRC) Dyspnea Scale [15], which has been found to be a valid measure of disability and predict mortality in COPD patients [16, 17] and the Memorial Symptom Assessment Scale, which has been found to predict mortality in lung cancer patients [18, 19] and correlates with quality-of-life measures in COPD [20]. The association between mortality and the Memorial Symptom Assessment Scale has not been examined for COPD or other chronic respiratory diseases. These PRO instruments may predict mortality in bronchiectasis and NTM, but their use has not been fully evaluated.

The National Institutes of Health (NIH) has an ongoing natural history cohort of bronchiectasis patients, and PRO instruments were systematically incorporated into these studies in 2015. Most patients in the cohort have a history of NTM pulmonary disease (NTM PD). The objective of this analysis was to evaluate predictors of short-term mortality in this cohort, including the 6MWT and PRO instruments. These data could help inform evaluations of prognosis in bronchiectasis patients for patient education and possibly referral to hospice care.

## Methods

### Study population

Our study population for this retrospective analysis included adults enrolled in the Institutional Review Board-approved natural history study of bronchiectasis at the National Institutes of Health (NIH), (www.clinicaltrials.gov identifier NCT00943514). Inclusion criteria are 5 years of age and older with an established diagnosis of bronchiectasis or a history of chronic, recurring respiratory infections. Patients could also be enrolled concurrently in a natural history study of NTM infections (www.clinicaltrials.gov identifier number NCT00018044), and for this reason the patient population is enriched for persons with NTM PD. Patients enrolled in these protocols have annual follow-up visits but may be seen more frequently at the discretion of the clinician. Enrollment and follow-up visits include a medical history and physical examination, pulmonary function tests (PFT), including spirometry, lung volumes and diffusing capacity, a 6MWT, and sputum (induced, if needed) microbiology for routine bacteria, acid fast bacilli, and fungal culture. In addition, beginning in January 2015, patients completed PRO instruments capturing HRQL and pulmonary symptom-specific questionnaires, including the SGRQ, the MRC Dyspnea Scale, and the Pulmonary Symptom Severity Scale (PSSS) (described below). For this reason, we defined January 1st, 2015 as our baseline date. Patients were included in our analytic cohort if they were aged ≥ 18 years old, had bronchiectasis, and had a 6MWT or SGRQ completed on or after this date. The end of the observation period was defined as December 31, 2019. We excluded patients diagnosed with a primary immunodeficiency and those whose baseline study visit was < 6 months prior to the end of the study period.

### Data abstraction

Demographic, clinical, microbiologic, radiographic, and PRO instrument data were abstracted from the NIH Clinical Center electronic medical records. Patient study baseline was defined as the first 6MWT or SGRQ after January 1st, 2015. Other baseline measurements (clinical, microbiologic, radiographic) were defined as the closest measurement within six months before or after the baseline to accommodate varying follow up. For study purposes, patient chest computed tomography (CT) scans were re-reviewed by two pulmonologists (KF, LG) to assess the presence of fibrocavitary disease.

Antibiotic treatment status at baseline was determined by querying clinical records for current prescriptions (NIH and outside institutions) on the date of baseline study visit for antibiotics of interest. A patient was considered on antibiotic treatment if records indicated active prescription, regardless of treatment duration or indication.

### Functional and quality of life measurements

The 6MWT measures the distance a patient is able to walk in 6 minutes (6MWD), providing a simple, objective measure of functional exercise capacity [21]. A sample of healthy adults aged 40–80 found a median 6MWD of 580 m for men and 500 m for women. Reference equations have been derived from several study populations [22–24]. We used reference equations for patients aged 45–85 years [25]. Estimates for a minimum important distance for 6MWT in the context of respiratory diseases are between 25 and 30 m [24, 26, 27]. The distance saturation product (DSP) adjusts for oxygen de-saturation by multiplying the 6MWD by the lowest oxygen saturation during the walk [21]. Available data included only pre-and post-walk oxygen saturation and for that reason we used post-walk oxygen saturation to calculate DSP. The SGRQ is a self-administered, HRQL questionnaire assessing a person’s perception of his or her breathing problems with regards to symptoms, activity, and impact [28]. Each section is scored from 0 to 100 and is weighted to calculate a total score also from 0 to 100, with higher scores indicating greater severity. A minimum number of responses are needed for the questionnaire to be considered valid. Published median scores in the general population are 8.2 for women and 8.6 for men [29]. The MRC Dyspnea Scale instructs patients to score their dyspnea severity on a scale of 1–5, with grade 5 indicating highest severity [15]. The PSSS was modified in-house from the Memorial Symptom Assessment Scale to include the frequency and severity of cough, shortness of breath, sputum production, fatigue, and wheezing. For each symptom a patient indicates is present, the frequency (PSSS-frequency) and severity (PSSS-severity) of each symptom is rated from 1 to 4, with 4 indicating the highest severity. A total score for symptom frequency and symptom severity is assigned from 1 to 20 (Additional file 1: Fig. S1).

### Survival analysis

For purposes of visualization, we estimated Kaplan–Meier survival curves with the 6MWD transformed by previously-published reference equations to the percent of distance walked predicted based on gender, height, and weight [25] and categorized as < 50%, 50–< 75%, and ≥ 75%. We assessed the difference in survival curves using a Mantel–Haenszel test with statistical significance defined as p < 0.05. We evaluated predictors of survival in both univariable and multivariable Cox proportional hazards models, with a focus on PRO and 6MWT measurements. We first constructed univariable models for all measured demographic, microbiologic, and clinical factors abstracted, including comorbidities, NTM culture positivity, NTM species isolated, and other concomitant lung infections (Table 1). Sex, age, BMI, fibrocavitary disease status, forced expiratory volume in one second (FEV_1_% predicted) and diffusion capacity of carbon monoxide (DLCO adj% predicted), C-reactive protein and *M. abscessus* isolated at baseline were significant.

**Table 1:** Baseline demographic and clinical characteristics of analytic cohort, bronchiectasis natural history study (n = 300)

Characteristic	N (%)
Follow up (years)–median [IQR]	4.1 [2.8–4.6]
Female	217 (72)
Age–median [IQR]	62 [50–69]
Died	21 (7)
Body Mass Index (kg/m)–median [IQR]	21.4 [19.8–24.2]
Fibrocavitary disease	44 (15)
FEV_1_% predicted–median [IQR]	72.5 [58–86.2]
DLCO adj% predicted–median [IQR]	64 [53.8–74]
C-reactive protein (mg/L)–median [IQR]	2.1 [0.8–6]
Comorbidities	
Asthma	57 (19)
Cystic fibrosis	20 (7)
Chronic obstructive pulmonary disease	12 (4)
Pulmonary hypertension	8 (3)
Primary ciliary dyskinesia	2 (1)
NTM positive since enrollment^b^	219 (74)
MAC^c^	168 (57)
*M. abscessus*^d^	97 (33)
Other NTM species	65 (22)
NTM positive at study baseline	150 (51)
MAC	96 (64)
*M. abscessus*	62 (41)
Other NTM species	24 (16)
AFB smear positive at study baseline	49 (17)
Non-NTM organisms at study baseline	
*Aspergillus*	91 (31)
*Pseudomonas*	53 (18)
*Staphylococcus aureus*	17 (6)
*Stenotrophomonas*	18 (6)

Missing data: BMI: n = 5, Fibrocavitary disease: n = 35, FEV_1_: n = 12, DLCO: n = 16, C-reactive protein: n = 1, Microbiology: 6 patients without pulmonary cultures taken at NIH (unable to produce sputum after induction, appointment no-show)

*IQR* interquartile range [25th percentile–75th percentile], *BMI* body mass index, *FEV*_1_ forced expiratory volume in 1 s, *DLCO* diffusing capacity of the lungs for carbon monoxide, *NTM* nontuberculous mycobacteria, *MAC* *Mycobacterium avium* complex, *AFB* Acid-fast bacilli

^a^Excludes *M. gordonae*

^b^Includes: *Mycobacterium avium complex, Mycobacterium avium, Mycobacterium intracellulare/chimaera, Mycobacterium chimaera, Mycobacterium intracellulare*

^c^Includes: *Mycobacterium abscessus*, *Mycobacterium abscessus* group, *Mycobacterium massiliense, Mycobacterium chelonae, Mycobacterium chelonae* group

We further considered these variables significant in univariable models for inclusion in our final multivariable models using likelihood ratio tests to evaluate model fit. All measures significant in univariable models significantly improved multivariable model fit, except C-reactive protein. The low number of deaths in this cohort during the study period necessitated efficient variable selection for the final model to avoid overfitting. We selected one measure of severity, fibrocavitary disease status, due to previous independent association with mortality in a similar cohort [30] and FEV_1_% predicted and DLCO adj% predicted were dropped from the multivariable model.

Our final model included *M. abscessus* status, age, sex, BMI, and fibrocavitary disease status, in addition to the PRO instruments, 6MWD, or DSP. Because of correlation among PRO and 6MWD, we constructed separate multivariable models for each PRO and 6MWT measurements. To control for different baseline mortality hazard between men and women, we constructed Cox models stratified by sex. All analyses were conducted using R version 4.0.2 survival and survminer packages. Total follow-up time was defined as the interval between the study baseline and the date of death or the end of the observation period, December 31st, 2019 for surviving patients.

## Results

### Baseline characteristics

A total of 300 patients were included in our analytic cohort, with a median of 4.1 years (range 39 days–4.98 years) of follow-up; cumulative follow-up time for the entire cohort was 1072 years. Baseline demographic, clinical, and microbiological features are shown in Table 1.

The median age at baseline was 62 years and 217 (72%) were women. The median BMI was 21.4 kg/m^2^, median FEV_1_% predicted was 72.5%, and 44 (15%) had fibrocavitary disease. The most common comorbidities were asthma (19%) and cystic fibrosis (7%). Twenty-one patients died for a crude mortality of 7% and a mortality rate of 2 per 100 person-years.

### Microbiology

Overall, 219 (74%) had a history of NTM isolation from respiratory culture collected at NIH since enrollment in the protocol, including 168 (57%) with MAC infections and 97 (33%) with *M. abscessus* infections. Of the 150 (51%) patients with NTM isolated at baseline, 96 (64%) had MAC, 62 (41%) had *M. abscessus*, and 24 (16%) had another NTM species (excluding *M. gordonae*). Baseline concomitant organisms included *Pseudomonas* in 53 (18%)*, Aspergillus* in 91 (31%), *Staphylococcus aureus* in 17 (6%), and *Stenotrophomonas* in 18 (6%) (Table 1).

The antibiotics received at baseline for the cohort are shown in Additional file 1: Table S1.

Among those with MAC infections, 45% were receiving treatment including a macrolide, and 17% were receiving a regimen that included amikacin. Among those with *M. abscessus* infections, 37% received a form of amikacin, and 21% received clofazimine. Overall, of 150 patients with NTM isolated within ± 6 months of baseline, 84 (56%) were receiving antibiotic treatment.

### Functional/PRO measurements

Functional and PRO measures are shown in Table 2.

**Table 2:** Functional and Quality of Life Measurements (n = 300)

Measurement	N (%)
6-min walk test–Median [IQR]	
6MWD (m)	505 [441.5–576.8]
Distance Saturation Product (m%)	479.8 [427.7–555.8]
St. George’s Respiratory Questionnaire–Median [IQR]	
Total Score	30 [15.8–48.2]
Symptom Score	44.5 [30.4–62.3]
Activity Score	36.4 [13–59.5]
Impact Score	23.3 [10.3–38.2]
Dyspnea Scale	
Grade 1	132 (44)
Grade 2	100 (33)
Grade 3	32 (11)
Grade 4	3 (1)
Grade 5	3 (1)
Pulmonary Symptom Severity Score–Median [IQR]	
Frequency	9 [5.2–11]
Severity	6 [4–8]

Missing data: 6MWD: n = 18, 6 MW DSP: 41, SGRQ: total score n = 20, symptom score n = 20, activity score n = 19, impact score n = 19, Dyspnea scale: n = 30, PSSS-severity: 40, PSSS-frequency: 36

*6MWD* 6-Minute walk distance, *IQR* interquartile range [25th percentile–75th percentile]

**Fig. 1:**
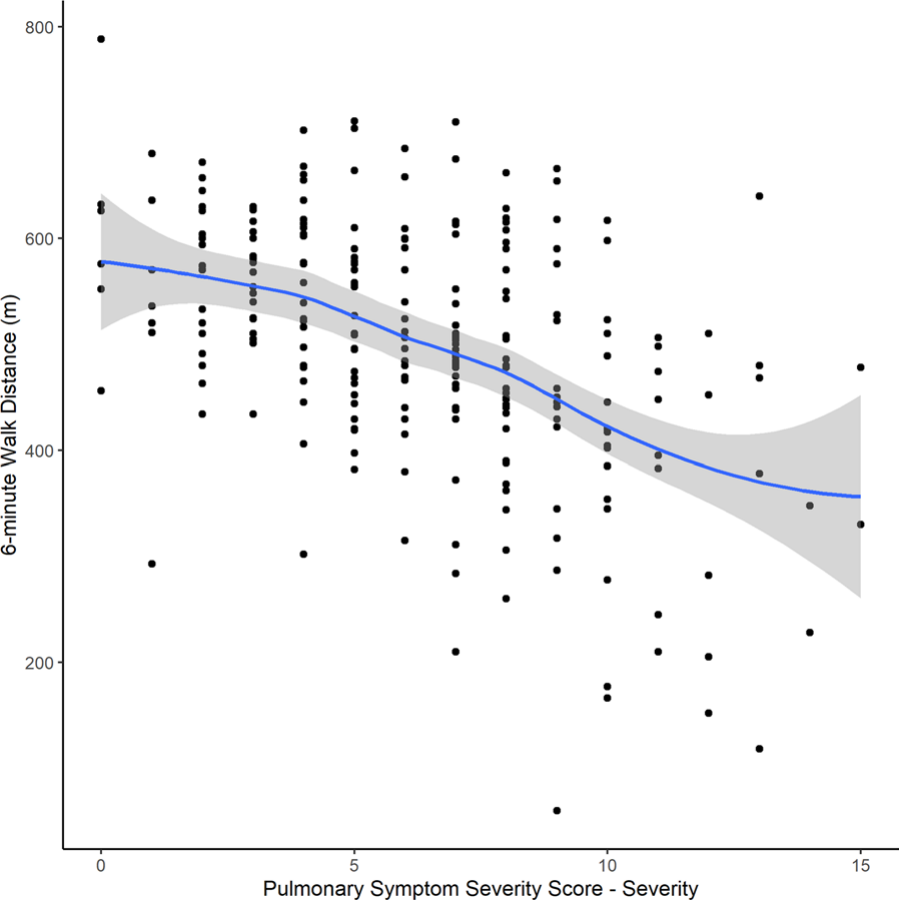
Correlation between the 6-minute walk distance and Pulmonary Symptom Severity Score-severity component. Correlation visualized by scatter plot with locally weighted scatter-plot smoother lines

The median distance walked was 524 m for men, 498 m for women, and 505 m overall. The median 6MWD percent predicted was 76% among those in the cohort age 45–85 years. The median DSP was 495 m% for men, 474 m% for women, and 480 m% overall. The median total SGRQ score was 30.0 points, more than threefold the median score in the general population, indicating poorer HRQL. For the component scores, the median symptom score was 44.5; activity- 36.4; impact- 23.3. The MRC Dyspnea Scale indicated generally mild dyspnea, with 232 (77%) patients selecting grade 1–2. The median PSSS-frequency score was higher than the median PSSS-severity score, at 8 and 6 respectively. All PRO measures were correlated with each other as well as with 6MWD (Table 3) with Spearman correlation coefficients significant at p < 0.001 (Fig. 1, Additional file 1: Fig. S2).

**Table 3:** Spearman correlation coefficients among PRO measures and 6MWD

	SGRQ Total Score	6MWD	MRC Dyspnea Scale
6MWD	− 0.53*	NA	NA
MRC Dyspnea Scale	0.6*	− 0.35*	NA
PSSS-severity	0.76*	− 0.46*	0.45*
PSSS-frequency	0.76*	− 0.4*	0.45*

*6MWD* 6-minute walk distance, *MRC* Medical Research Council, *SGRQ* St. George’s Respiratory Questionnaire, *PSSS* Pulmonary Symptom Severity Score (contains two components -severity and -frequency)

*Significant at p < 0.001

The strength of correlations varied, with a high positive correlation between the SGRQ and MRC Dyspnea scale and PSSS, a moderate negative correlation between the SGRQ and 6MWD, low negative correlations between the 6MWD and MRC Dyspnea scale and PSSS, and low positive correlation between the MRC Dyspnea scale and the PSSS.

### Survival analysis

**Fig. 2:**
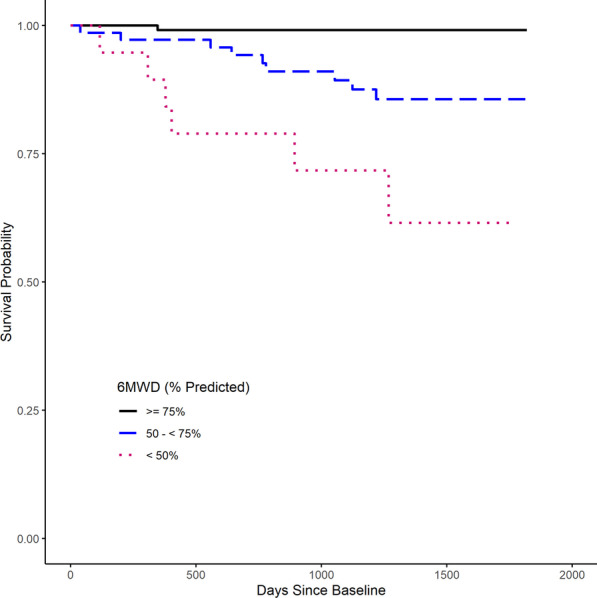
Kaplan–Meier survival curves. Percent predicted 6-minute walk distance (6MWD) for patients age 45–85 Curves significant at p < 0.001 as determined by Mantel–Haenszel test

Kaplan–Meier survival curves are presented in Fig. 2 for the 6MWD. Survival probability decreased significantly as the percent predicted 6MWD decreased.

The SGRQ, MRC Dyspnea Scale, and PSSS-frequency were not independently predictive of mortality after controlling for age, sex, BMI, fibrocavitary disease status, and *M. abscessus* infection. In multivariable models, 6MWD, DSP, and the PSSS-severity were all independently predictive of mortality. The adjusted hazard ratios for final models are shown in Table 4. Un-adjusted hazard ratios and hazard ratios for variables included to control confounded may be found in Additional file 1: Table S2.

**Table 4:** Cox proportional hazard final models

	Adjusted hazard ratio (95% CI)	p value
6MWD (10 m)^b^	0.938 (0.896–0.981)	0.005
6 MW DSP (10 m%)^b^	0.930 (0.887–0.974)	0.002
PSSS-severity^b^	1.29 (1.04–1.59)	0.02
BMI (kg/m^2^)^c^	0.589 (0.469–0.739)	< 0.001

^a^6MWD: n = 242, n events = 17; 6 MW DSP: n = 222, n events = 17; PSSS-severity: n = 232, n events = 18; BMI: n = 257, n events = 19

^b^Model adjusted for: age, BMI, Fibrocavitary disease, *M. abscessus*, cox regression stratified by gender

^c^Model adjusted for: age, Fibrocavitary disease, *M. abscessus*, cox regression stratified by gender

*CI*= confidence interval, *BMI* body mass index, *6MWD* 6-minute walk distance, *6MW DSP* 6-minute walk distance saturation product, *PSSS-severity* Pulmonary Symptom Severity Score severity component

The 6MWD and DSP standardized to units of 10 m and 10 m% were found to have an adjusted hazard ratio of 0.94 and 0.93, respectively, corresponding to a 6% reduction in risk of death per 10 m and a 7% reduced risk per 10 m% walked. Translated into units of the upper estimate of the minimum important difference, the hazard ratio was 0.83 (95% CI 0.74–0.94) or a 17% reduced risk of death per 30 m walked for the 6MWD. The adjusted hazard ratio of the PSSS severity score was 1.29: for each unit increase in the severity score, the risk of death increased by 29%. Additionally, after stratifying by sex and controlling for age, fibrocavitary disease, and *M. abscessus* we found an increased risk of mortality with decreasing BMI. For each 1 kg/m^2^ decrease in baseline BMI, the risk of death increased by 41%.

## Discussion

We found that the PSSS-severity (but not frequency), 6MWD and the DSP were independently predictive of mortality after controlling for age, fibrocavitary disease, *M. abscessus*, BMI, and sex. These findings suggest that a PRO instrument capturing pulmonary symptom severity and functional measures may add to the assessments of a patient’s clinical status and risk of mortality, beyond previously described risk factors in bronchiectasis and NTM [6].

The independent effects of the 6MWD and the DSP are noteworthy and highlight the value of this relatively straightforward measurement. This study builds on research demonstrating the clinical utility of the 6MWT for pulmonary diseases. In a study of idiopathic bronchiectasis with 60 patients and 9 deaths, the DSP was the strongest 6MWT measurement in predicting mortality [31]. The 6MWD is a measure of gait speed, which has been found to be predictive of mortality across disease conditions: a systematic review across nine large cohort studies of older adults with conditions ranging from osteoporosis to healthy aging, found that gait speed was predictive of mortality independent of all other measures. Slower gait speed may reflect decline of the many organ systems required to walk, including the respiratory system, which in turn are associated with mortality [32]. Thus, 6MWD captures an effect that is important independent of other measures. The DSP adds a measure of respiratory system strain by adjusting for oxygen desaturation during the walk. However, the similarity of adjusted hazard ratios between the 6MWD and DSP is notable for clinical practice, suggesting clinical utility of the 6MWT even if oxygen saturation monitoring is not available.

The predictive value of the PSSS-severity for mortality in this patient population highlights the clinical utility of this measure, especially given the advantages of PRO instruments including low patient and resource burden, ability to be completed on first assessment, and ease of tracking longitudinally. This measure could be especially useful when disease severity contraindicates or limits a patient’s willingness to complete a 6MWT or other field walking tests. However, this instrument has not been systematically validated as a modification of the Memorial Symptom Assessment Scale and future verification of the reliability and validity in other patient populations is warranted. The association of the PSSS-severity with survival suggests a patient’s perception of severe pulmonary symptoms correlates with disease progression and respiratory system decline. Even though the SGRQ, MRC Dyspnea Scale, and PSSS-frequency are correlated with the PSSS-severity, we did not find these instruments were significantly associated with mortality risk in multivariable models in this patient population, although an SGRQ score ≥ 25 was recently found to predict mortality in an MAC-PD cohort after adjusting for age, BMI, and percent predicted forced vital capacity [33]. It is possible that NTM-specific PRO instruments would better predict mortality in this patient population. A recent assessment of a newly-developed NTM-specific PRO questionnaire found that a module assessing NTM-specific domains performed well, in addition to the previously validated Quality of Life-Bronchiectasis (QOL-B), lending validity to the need for specific NTM PRO instruments [34].

BMI has also been found to predict increased risk of disease as well as risk of death in other bronchiectasis as well as NTM studies. Among patients in the bronchiectasis research registry, underweight patients had lower lung function measured by FEV_1_% predicted [35]. In a separate recent study, low BMI was also correlated with increased disease severity (Bronchiectasis Severity Index), QOL (QoL-B), and symptoms (Leicester Cough Questionnaire) [36]. A large cohort study of more than 5.6 million persons in the South Korean national health care system who had regular pulmonary exams found that decreasing BMI was inversely related to the risk of NTM PD [37]. Relevant to increased risk of NTM PD among underweight bronchiectasis patients, low BMI was found to be associated with treatment failure in patients with *M. abscessus* pulmonary infections [38] and a study in Japan found that NTM PD patients who died had significantly lower BMI than surviving patients [39]. Most recently, a large cohort study also found BMI to be predictive of mortality in NTM PD patients in the context of the newly developed score which incorporates body mass index, age, cavity, erythrocyte sedimentation rate, and sex (BACES) into a predictive model [40].

Hospice care may be under-utilized for pulmonary conditions and uncertainty regarding prognosis may be a contributing factor [41]; for this reason, adding to the understanding of predictors of prognosis among bronchiectasis and NTM patients could be useful for patient education and end-of-life care [42]. Our findings suggest that irreversible weight loss could be a factor to consider in hospice referral decision-making, consistent with the Medicare hospice benefit criteria for pulmonary disease [41]. Additionally, the 6MWD and PSSS-severity could be useful documentation for the severe chronic lung disease component of this criteria, which requires dyspnea resulting in decreased functional capacity.

A limitation of our study is that the patient population was referred to our tertiary care center and thus are more likely to represent patients with severe disease and may not be representative of all patients with bronchiectasis or NTM PD. We are also unable to determine if low BMI is a consequence of worsening disease or is a cause of disease progression.

## Conclusions

Our study provides evidence that the PSSS-severity PRO instrument and the 6MWD and DSP are independently associated with bronchiectasis mortality, and that underweight patients are at higher risk of mortality. Further studies are needed to fully understand the impact of weight gain or improvement of functional capacity on outcomes, and these studies should incorporate PROs specific to bronchiectasis and NTM PD.

## Supplementary Information

**Additional File 1: Figure S1.** Pulmonary Symptom Severity Score questionnaire. **Figure S2.** Correlations visualized between St. George’s Respiratory Questionnaire and (a) six-minute walk distance; (b) Pulmonary Symptom Severity Score (PSSS)-severity; (c) PSSS-frequency; Medical Research Council Dyspnea Scale and (d) St. George’s Respiratory Questionnaire; (e) 6-minute walk distance; (f) PSSS-severity; (g) PSSS-frequency; (h) 6-minute walk distance and PSSS-frequency. **Table S1.** Antibiotic Treatment at Baseline (n (%)). **Table S2.** Cox proportional hazard final models.

## Data Availability

The de-identified dataset for this analysis is available from the corresponding author on reasonable request and consultation with the NIH Office of Human Subjects Research Protection.

## Abbreviations

NTM: Nontuberculous mycobacteria
PRO: Patient reported outcome
6MWT: 6-Minute walk test
SGRQ: St. George’s Respiratory Questionnaire
PSSS: Pulmonary Symptom Severity Score
BMI: Body mass index
MAC: *Mycobacterium avium* Complex
COPD: Chronic obstructive pulmonary disease
HRQL: Health-related quality of life
MRC: Medical Research Council
PD: Pulmonary Disease
NIH: National Institutes of Health
PFT: Pulmonary function test
CT: Computed tomography
6MWD: 6-Minute walk distance
DSP: Distance saturation product
PSSS-frequency: Pulmonary Symptom Severity Score Frequency Score
PSSS-severity: Pulmonary Symptom Severity Score Severity Score
FEV_1_% predicted: Percent predicted forced expiratory volume in one second
DLCO adj% predicted: Percent predicted diffusion capacity of carbon monoxide
IQR: Interquartile range [25th percentile–75th percentile]
AFB: Acid-fast bacilli
QOL-B: Quality of Life-Bronchiectasis
BACES: Body mass index, age, cavity, erythrocyte sedimentation rate, and sex
