# Supplementary material for: The 6-minute walk test predicts mortality in a pulmonary nontuberculous mycobacteria-predominant bronchiectasis cohort
Source: BMC Infect Dis. 2022 Jan 21;22:75. doi: 10.1186/s12879-022-07054-6 (PMC8783466; doi:10.1186/s12879-022-07054-6)
Supplement: Supplementary file 1 — Additional File 1: Figure S1. Pulmonary Symptom Severity Score questionnaire. Figure S2. Correlations visualized between St. George’s Respiratory Questionnaire and (a) six-minute walk distance; (b) Pulmonary Symptom Severity Score (PSSS)-severity; (c) PSSS-frequency; Medical Research Council Dyspnea Scale and (d) St. George’s Respiratory Questionnaire; (e) 6-minute walk distance; (f) PSSS-severity; (g) PSSS-frequency; (h) 6-minute walk distance and PSSS-frequency. Table S1. Antibiotic Treatment at Baseline (n (%)). Table S2. Cox proportional hazard final models. [file 12879_2022_7054_MOESM1_ESM.pdf]

PULMONARY SYMPTOM SEVERITY SCORE
Protocol 09-H-0172

NAME: DATE:

INSTRUCTIONS**: We have 5 symptoms below. *If you have had any of these in the last week* please note; "HOW OFTEN" and "HOW SEVERE", by circling the appropriate number; select only one number. If you "DID NOT HAVE" the symptom make an X in the box marked "DID NOT HAVE"**

**DURING THE PAST WEEK.** DID YOU HAVE ANY OF THESE?	DID NOT HAVE	**IF YES HOW OFTEN DID YOU HAVE IT** RARELY	OCCASIONALLY	FREQUENTLY	ALMOST CONSTANTLY	**IF YES HOW SEVERE WAS IT USUALLY** SLIGHT	MODERATE	SEVERE	VERY SEVERE
COUGH		**1**	**2**	**3**	**4**	**1**	**2**	**3**	**4**
SHORTNESS OF BREATH		**1**	**2**	**3**	**4**	**1**	**2**	**3**	**4**
SPUTUM PRODUCTION		**1**	**2**	**3**	**4**	**1**	**2**	**3**	**4**
FATIGUE		**1**	**2**	**3**	**4**	**1**	**2**	**3**	**4**
WHEEZING		**1**	**2**	**3**	**4**	**1**	**2**	**3**	**4**

Supplementary Figure 1: Pulmonary Symptom Severity Score questionnaire

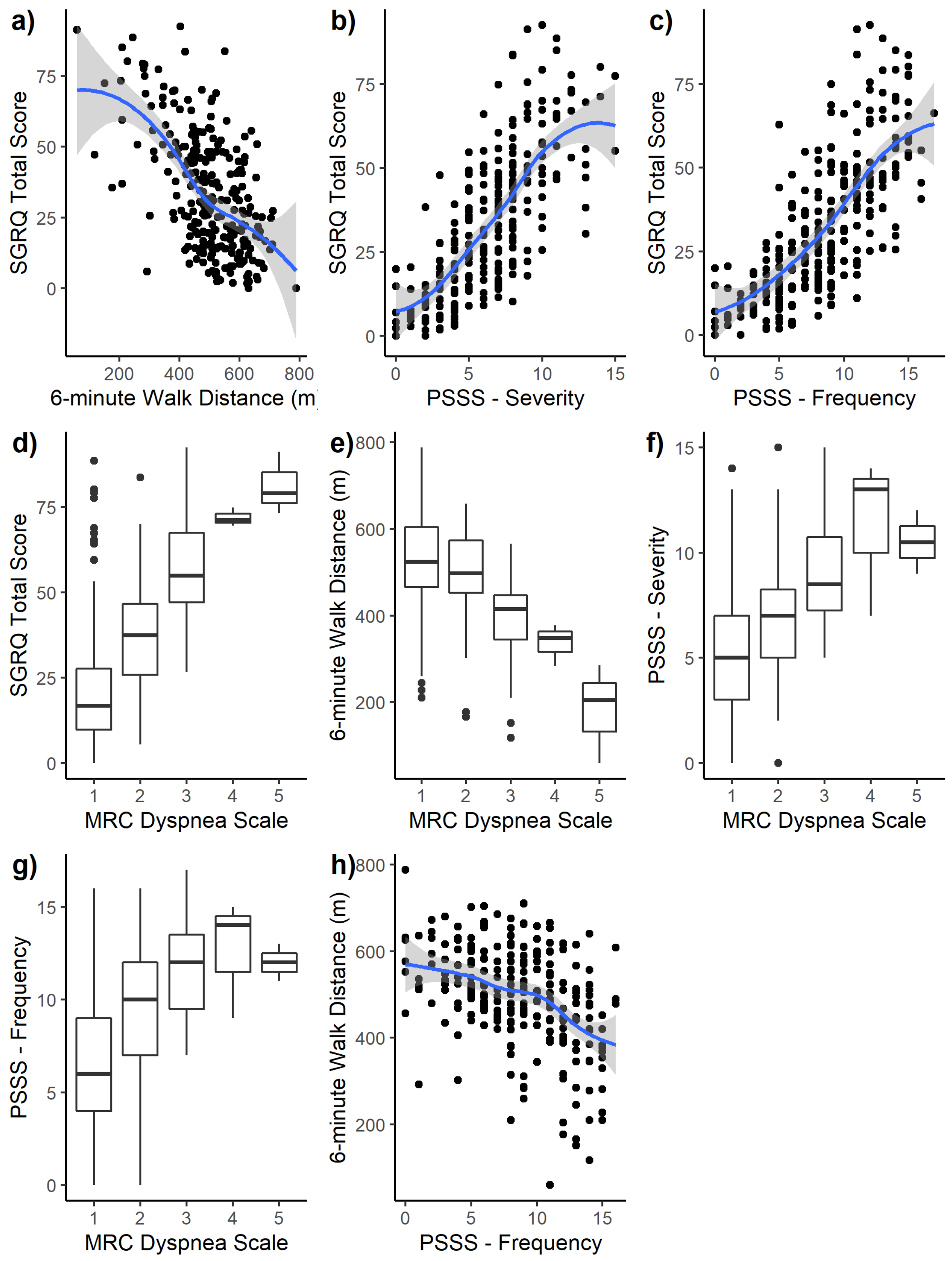

Supplementary Figure 2: Correlations visualized between St. George's Respiratory Questionnaire and **a)** six-minute walk distance; **b)** Pulmonary Symptom Severity Score (PSSS)-severity; **c)** PSSS-frequency; Medical Research Council Dyspnea Scale and **d)** St. George's Respiratory Questionnaire; **e)** 6-minute walk distance **f)** PSSS-severity; **g)** PSSS-frequency; **h)** 6-minute walk distance and PSSS-frequency

Supplementary Table 1. Antibiotic Treatment at Baseline (n (%))

Antibiotic	Whole Cohort (n=300)	MAC (n=96)	*M. abscessus* (n=62)	Other NTM Species* (n=24)
Any NTM-active Antibiotic	152 (51)	51 (53)	37 (60)	10 (42)
Amikacin	49 (16)	16 (17)	23 (37)	4 (17)
Azithromycin	112 (37)	38 (40)	26 (42)	8 (33)
Clarithromycin	17 (6)	5 (5)	3 (5)	2 (8)
Clofazimine	24 (8)	9 (9)	13 (21)	0 (0)
Ethambutol	70 (23)	32 (33)	5 (8)	5 (21)
Imipenem/Cilastatin	10 (3)	2 (2)	8 (13)	0 (0)
Linezolid	21 (7)	7 (7)	14 (23)	0 (0)
Moxifloxacin	17 (6)	8 (8)	5 (8)	1 (4)
Rifabutin	8 (3)	3 (3)	0 (0)	1 (4)
Rifampin	55 (18)	26 (27)	5 (8)	6 (25)

* Excludes *M. gordonae*

Abbreviations: NTM = nontuberculous mycobacteria, MAC = *Mycobacterium avium* complex

Supplementary Table 2. Cox proportional hazard final models

	Unadjusted Hazard Ratio (95% CI)	Adjusted Hazard Ratio (95% CI)		
		Model 1	Model 2	Model 3
Age (years)	1.06 (1.02-1.10)	1.06 (1.01-1.1)	1.05 (1.01-1.1)	1.09 (1.04-1.15)
Male sex	2.51 (1.07-5.92)	-	-	-
BMI (kg/m^2)	0.694 (0.590-0.818)	0.642 (0.493-0.836)	0.654 (0.501-0.854)	0.622 (0.489-0.792)
Fibrocavitary disease	7.89 (3.12-19.64)	2.95 (0.973-8.94)	2.7 (0.893-8.15)	2.16 (0.701-6.64)
M. abscessus	3.54 (1.50-8.33)	5.89 (1.92-18)	6.4 (2.06-19.9)	4.18 (1.42-12.3)
6MWD (10 m)	0.903 (0.874-0.933)	0.938 (0.896-0.981)	-	-
6MW DSP (10 m)	0.898 (0.868-0.929)	-	0.93 (0.887-0.974)	-
PSSS-severity	1.22 (1.07-1.39)	-	-	1.29 (1.04-1.59)

Sample size and number of events - Unadjusted: Age, sex: n= 300, n events = 21; BMI: n = 295, n events = 21; fibrocavitary disease: n = 265, n events = 19; M. abscessus: n = 294, n events = 21; 6MWD: n = 282, n events = 19; 6MW DSP: n = 259, n events = 19; PSSS-severity: n = 260, n events = 20. Adjusted: Model 1: n= 242, n events= 17; Model 2: n= 222, n events = 17; Model 3: n = 227, n events = 18

Abbreviations: BMI = body mass index, 6MWD = 6-minute walk distance, 6MW DSP = 6-minute walk distance saturation product, PSSS-severity = Pulmonary Symptom Severity Score severity component
